# Mechanistic study of ECM phenotype switch in epicardial adipose tissue in atrial fibrillation: A bioinformatics study

**DOI:** 10.1097/MD.0000000000051461

**Published:** 2026-11-06

**Authors:** Huanmei Du, Xu Yang, Yankai Guo, Zukela Tuerhong, Jiasuoer Xiaokereti, Yujiao Zhang, Shirui Li, Jianghua Zhang

**Affiliations:** a Cardiac Pacing and Electrophysiology Department, The First Affiliated Hospital of Xinjiang Medical University, Urumqi, China; b Xinjiang Key Laboratory of Cardiac Electrophysiology and Cardiac Remodelling, The First Affiliated Hospital of Xinjiang Medical University, Urumqi, China.

**Keywords:** atrial fibrillation, COL4A4, collagen, epicardial adipose tissue, extracellular matrix, phenotype switch, ribosome biogenesis

## Abstract

Atrial fibrillation (AF) is the most common cardiac arrhythmia. Epicardial adipose tissue (EAT) is closely associated with AF, but its molecular mechanisms remain incompletely understood. This study aimed to investigate the extracellular matrix (ECM) remodeling characteristics of AF-related EAT. Using the publicly available RNA-sequencing dataset GSE135445 (AF: *n* = 6, sinus rhythm: *n* = 6), we performed differential expression analysis (DESeq2) on gene-level counts regenerated from the raw sequencing reads, single-gene analysis focusing on ECM gene families, and gene set enrichment analysis. An “ECM phenotype switch” hypothesis was proposed based on the coordinated dysregulation of interstitial and basement membrane collagen genes. Differential expression analysis identified 610 differentially expressed genes. Notably, the basement membrane-specific collagen COL4A4 was significantly upregulated in AF (log2FC = 4.37, *P*_adj_ = 2.7 × 10^−9^), accompanied by significant upregulation of COL4A3, whereas interstitial collagen repair genes (*COL1A1*, *COL1A2*, *COL3A1*, and *FN1*) showed consistent downregulation, defining an “ECM phenotype switch” characterized by suppressed interstitial repair coupled with independent basement membrane deposition; the fibrinolysis inhibitor SERPINE1 (plasminogen activator inhibitor-1) was also markedly upregulated (log2FC = 3.89, *P*_adj_ = .026). Gene set enrichment analysis implicated ribosome biogenesis and translation dysregulation as a potential associated upstream program (normalized enrichment score = 2.26–3.03 and false discovery rate = 0.012–0.013). AF-related EAT exhibits an ECM phenotype switch characterized by suppressed interstitial collagen repair and independent basement membrane collagen deposition, potentially associated with ribosome biogenesis dysregulation. These hypothesis-generating findings provide a novel framework for understanding EAT-mediated atrial remodeling in AF.

## 1. Introduction

Atrial fibrillation (AF) is the most common cardiac arrhythmia worldwide, with a prevalence of approximately 1% to 2%, and increases markedly with advancing age.^[1]^ AF is associated with substantial morbidity, including stroke, heart failure, and increased mortality, imposing a significant burden on global healthcare systems.^[2]^ Despite advances in catheter ablation techniques, including radiofrequency, cryoballoon, and pulsed field ablation, long-term recurrence rates remain high, largely because of incomplete understanding of the molecular mechanisms underlying AF initiation and maintenance.^[3]^

In recent years, the relationship between epicardial adipose tissue (EAT) and AF has emerged as a major focus of cardiovascular research. EAT is a fat depot located between the myocardium and the visceral pericardium, anatomically contiguous with the atrial myocardium without fascial separation. It is highly vascularized and innervated, and can directly influence adjacent cardiomyocytes through paracrine and endocrine signaling pathways.^[4]^ Numerous clinical studies have demonstrated that increased EAT volume is significantly associated with AF risk and postablation recurrence^.[5,6]^ However, the molecular mechanisms by which EAT promotes AF remain incompletely elucidated.

Extracellular matrix (ECM) remodeling is a central pathological feature of atrial structural remodeling in AF. The ECM comprises interstitial collagens (primarily types I and III), basement membrane collagens (type IV), proteoglycans, and glycosaminoglycans, which maintain the structural integrity and mechanical function of myocardial tissue.^[7]^ During AF progression, significant alterations in ECM composition occur, including increased interstitial fibrosis and basement membrane disruption, leading to increased myocardial stiffness and heterogeneous electrical conduction, thereby promoting AF maintenance.^[8]^

EAT as a metabolically active tissue adjacent to the atrial myocardium, its ECM remodeling status may directly influence the progression of atrial fibrosis. However, systematic investigations of ECM remodeling in AF-related EAT are scarce, and the coordinated regulation of different collagen subtypes (interstitial vs basement membrane) remains poorly understood. In this study, we utilized bioinformatics approaches based on the publicly available Gene Expression Omnibus dataset GSE135445 to systematically analyze transcriptomic differences between AF and sinus rhythm (SR) patients in EAT, with a focus on ECM remodeling characteristics. We propose the “ECM phenotype switch” hypothesis to provide new insights into AF prevention and treatment.

## 2. Materials and methods

### 2.1. Data source

This study utilized the publicly available dataset GSE135445 from the NCBI Gene Expression Omnibus database.^[9]^ This dataset contains RNA-sequencing data from 12 EAT samples (platform: Illumina HiSeq 4000), including 6 AF cases and 6 SR controls (Table 1). The original study describing this dataset has been published.^[10]^ All samples were obtained from patients undergoing cardiac surgery. The AF group comprised patients with persistent nonvalvular AF, and the SR group comprised patients with SR and no history of AF. Raw sequencing reads of the 12 samples were obtained from the NCBI Sequence Read Archive (SRA study SRP217534) and quantified with Salmon (v2.8.0) against the GENCODE v33 human transcript reference. Transcript-level abundances were summarized to gene-level counts with tximport (lengthScaledTPM), yielding a matrix of 60,662 genes × 12 samples. This study is a secondary analysis of publicly available, deidentified data; ethical approval and informed consent were obtained in the original study^[10]^, and no additional institutional review board approval was required for this secondary analysis.

**Table 1:** Basic sample information of the GSE135445 epicardial adipose tissue cohort.

Characteristic	AF group (n = 6)	SR group (n = 6)
Tissue source	Epicardial adipose tissue	Epicardial adipose tissue
Diagnosis	Persistent nonvalvular AF	Sinus rhythm
Sample size	6	6

Characteristics of the 12 EAT samples from the publicly available GEO dataset GSE135445, comprising 6 patients with persistent nonvalvular AF and 6 SR controls. The sample size in each group is indicated. Detailed clinical information (age, sex, and comorbidities) was not publicly available in the GEO record.

AF = atrial fibrillation, EAT = epicardial adipose tissue, GEO = Gene Expression Omnibus, SR = sinus rhythm.

### 2.2. Data preprocessing and differential expression analysis

All analyses were performed using R (version 4.3.0) and Bioconductor. Gene-level raw count data were obtained by transcript quantification of the raw sequencing reads as described in section 2.1. Differential expression analysis was performed using the DESeq2 package.^[11]^ The design formula was ~ group, where group was a factor with 2 levels (AF and SR). Differentially expressed genes were identified using the criteria: |log2FoldChange| > 1 and adjusted *P*-value (Benjamini–Hochberg false discovery rate [FDR]) < 0.05. Variance stabilizing transformation was applied for downstream visualization.

### 2.3. ECM-focused single-gene analysis

To systematically investigate ECM remodeling patterns in AF-related EAT, we curated a panel of 32 ECM-related genes stratified into 2 functional categories: interstitial collagen repair genes (*COL1A1*, *COL1A2*, *COL3A1*, *COL5A1*, *COL5A2*, *FN1*, *POSTN*, *VIM*, *ACTA2*, *TAGLN*, *MMP2*, *MMP9*, *MMP14*, *TIMP1*, *TIMP2*, *TGFB1*, *TGFB2*, *TGFB3*, *SERPINE1*, and *SPARC*) associated with fibrillar matrix maintenance; and basement membrane collagen genes (*COL4A1–COL4A6*, *LAMA2*, *LAMA4*, *LAMB1*, *LAMB2*, *NID1*, and *HSPG2*) associated with epithelial barrier integrity. Genes were required to be reliably detected in the count matrix, defined as at least 5 raw counts in at least 6 of the 12 samples; all 32 curated ECM genes met this criterion, and the per-gene detection counts are listed in Table S1, Supplemental Digital Content 1. Expression levels were extracted from the variance-stabilizing transformation–normalized matrix and compared between AF and SR groups using an independent samples *t*-test. Significance stars were annotated on boxplots for visual interpretation.

### 2.4. Gene set enrichment analysis

Gene set enrichment analysis (GSEA) was performed using the fgsea package^[12]^ based on the ranked gene list (sorted by DESeq2 Wald statistic), following the GSEA algorithm described by Subramanian et al^[13]^ Gene set permutations (n = 10,000) were performed using Gene Ontology (GO) Biological Process gene sets (C5; locally built from GO.db and homologous to MSigDB C5 GO:BP) and Kyoto Encyclopedia of Genes and Genomes (KEGG) pathways (C2; from the KEGG REST API), covering key biological processes including ribosome biogenesis, ribosomal RNA (rRNA) processing, messenger RNA (mRNA) splicing and processing, translation, protein folding, and ECM organization. Normalized enrichment score (NES), nominal *P*-value, and FDR were computed for each pathway. Statistical significance was defined as FDR < 0.25.

### 2.5. Statistical analysis

All statistical analyses were performed in R. Continuous variables are presented as mean ± SD. Between-group comparisons were performed using independent samples *t*-tests. GSEA significance was assessed by permutation testing. A 2-tailed *P* < .05 was considered statistically significant. To evaluate the robustness of the core findings to individual samples, a leave-one-out (jackknife) sensitivity analysis was additionally performed: the DESeq2 AF-versus-SR contrast was repeated 12 times, each iteration excluding 1 sample, and the log2 fold change, nominal *P*-value, and Benjamini–Hochberg-adjusted *P*-value of 6 key ECM genes (*COL4A4*, *COL1A1*, *COL1A2*, *COL3A1*, *FN1*, and *SERPINE1*) were recorded (Table S2, Supplemental Digital Content 2).

Note on analytical strategy: Initial exploratory analysis included weighted gene co-expression network analysis. However, given the limited sample size (n = 12), module-trait associations did not reach statistical significance (*P* > .05). Weighted gene co-expression network analysis typically requires larger cohorts (n > 20) for reliable inference. Therefore, we transitioned to a single-gene analysis approach with ECM-focused gene curation, ensuring methodologically sound conclusions within the constraints of the available data.

## 3. Results

### 3.1. Differential expression analysis

Differential expression analysis identified 610 differentially expressed genes, including 330 upregulated and 280 downregulated genes (Fig. 1)

**Figure 1.**
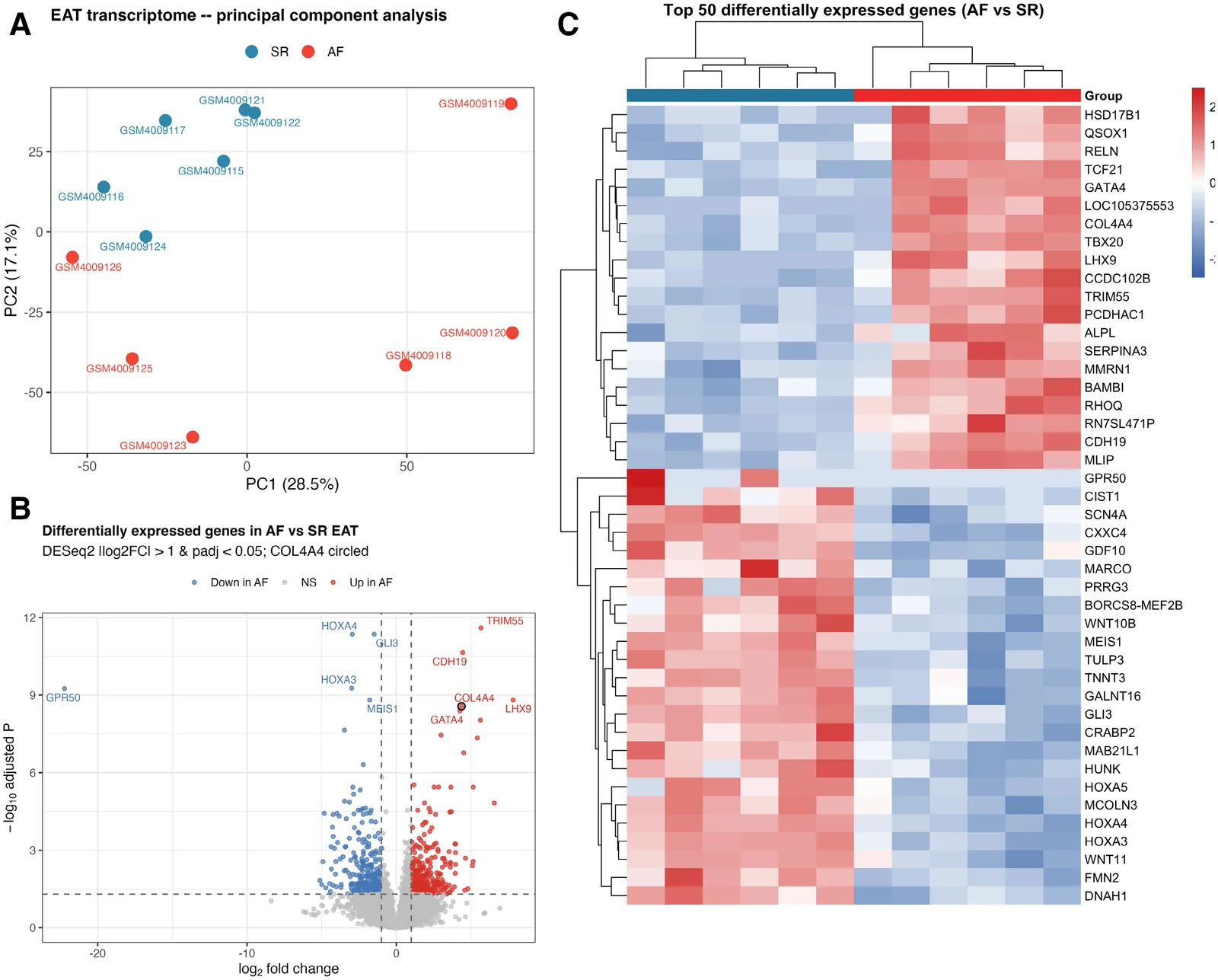
EAT transcriptome analysis overview. (A) PCA of EAT transcriptome data from 6 SR and 6 AF patients, showing clear separation between groups along PC1. (B) Volcano plot of DEGs between AF and SR EAT, with upregulated genes in red and downregulated genes in blue (adjusted *P* < .05, |log2FC| > 1). COL4A4 is prominently annotated in the significantly upregulated region. (C) Heatmap of the top 50 DEGs, revealing distinct expression patterns between AF and SR samples. AF = atrial fibrillation, DEG = differentially expressed gene, EAT = epicardial adipose tissue, FC = fold change, PCA = principal component analysis, SR = sinus rhythm.

### 3.2. ECM phenotype switch in AF-related EAT

To dissect the ECM remodeling architecture of AF-related EAT, we performed single-gene analysis on the curated ECM gene panel. All 32 curated ECM genes were reliably detected in the regenerated count matrix (detection criterion: ≥5 counts in ≥6 samples; Table S1, Supplemental Digital Content 1) and were included in the expression profiling.

Strikingly, COL4A4 (basement membrane collagen IV alpha-4 chain) was significantly upregulated in the AF group (log2FC = 4.37, *P*_adj_ = 2.7 × 10^−9^; Fig. 2A), consistent with its prominent position in the volcano plot. In contrast, interstitial collagen repair genes *COL1A1* (log2FC = −1.73), *COL1A2* (−0.94), *COL3A1* (−0.77), and *FN1* (−1.22) were consistently downregulated in AF (Fig. 2B), while the fibrinolysis inhibitor SERPINE1 (PAI-1) was markedly and significantly upregulated (log2FC = 3.89, *P*_adj_ = .026). This dissociation – suppressed interstitial collagen repair coupled with upregulated basement membrane collagen deposition – defines an “ECM phenotype switch” in AF-related EAT. The heatmap of the top 20 differentially expressed genes further illustrates the distinct expression patterns between groups (Fig. 2C). The robustness of this pattern was confirmed by the leave-one-out sensitivity analysis: after exclusion of each sample in turn, COL4A4 remained strongly and significantly upregulated (log2FC = +4.18 to +4.63; *P*_adj_ < 3 × 10^−7^ in all 12 iterations), whereas *COL1A1*, *COL1A2*, *COL3A1*, and *FN1* remained consistently downregulated (log2FC < 0 in every iteration) and SERPINE1 remained upregulated (log2FC = +3.26 to +4.19) (Table S2, Supplemental Digital Content 2).

**Figure 2.**
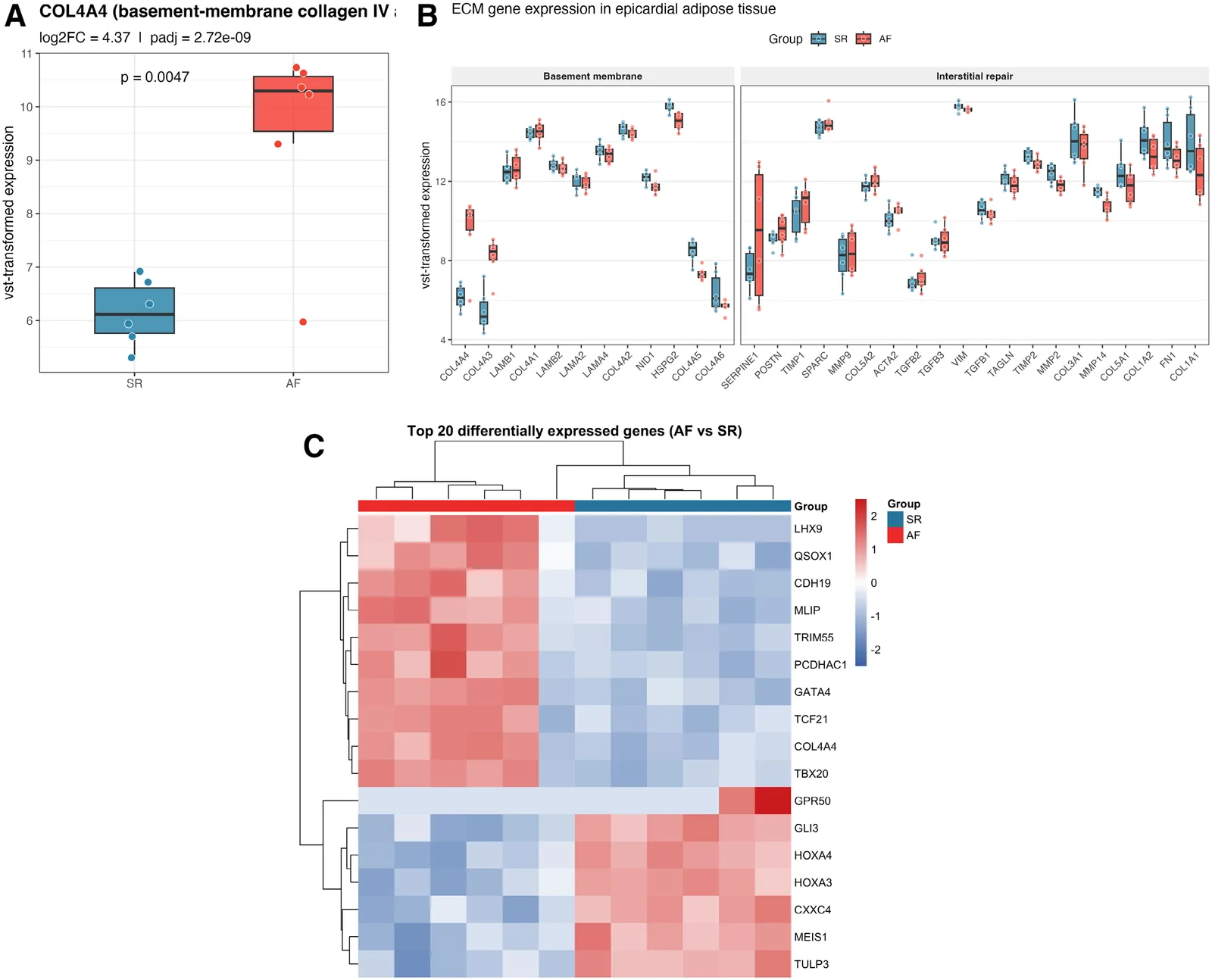
ECM phenotype switch in AF-related EAT. (A) Boxplot of COL4A4 expression levels in EAT from SR and AF patients, showing significant upregulation in AF (DESeq2; *P* < .01). (B) ECM gene panel expression comparing interstitial collagen repair genes (left) versus basement membrane collagen genes (right) between AF and SR groups. Significance indicated by stars (*P < .05, **P < .01, ***P < .001). (C) Heatmap of top 20 differentially expressed genes showing distinct expression patterns between groups. AF = atrial fibrillation, EAT = epicardial adipose tissue, ECM = extracellular matrix, SR = sinus rhythm.

Table 2 presents the detailed expression statistics for the detected ECM genes. Notably, COL4A4 ranked ninth among all differentially expressed genes and fourth among upregulated genes by adjusted *P*-value (*P*_adj_ = 2.7 × 10^−9^; Table 3), underscoring its prominence in the AF transcriptomic signature. The ECM phenotype switch pattern suggests a shift from adaptive interstitial remodeling to pathological basement membrane barrier disruption in AF-related EAT.

**Table 2:** Expression statistics of the curated extracellular matrix genes in AF versus SR epicardial adipose tissue.

Category	Gene	log2FC	*P*_adj_	Interpretation
Basement membrane (up)	*COL4A4*	+4.37	2.72e−09	Pathological deposition
Basement membrane (up)	*COL4A3*	+3.20	.00207	Independent upregulation
Fibrinolysis inhibition (up)	*SERPINE1*	+3.89	.0261	PAI-1 upregulation; impaired fibrinolysis
Interstitial repair (down)	*COL1A1*	−1.73	.159	Repair suppression (down, NS)
Interstitial repair (down)	*COL1A2*	−0.94	.279	Repair suppression (down, NS)
Interstitial repair (down)	*COL3A1*	−0.77	.506	Repair suppression (down, NS)
Interstitial repair (down)	*COL5A1*	−0.84	.351	Repair suppression (down, NS)
Interstitial repair (down)	*FN1*	−1.22	.16	Matrix disassembly (down, NS)
Basement membrane (NS)	*COL4A2*	−0.27	.488	NS
Basement membrane (NS)	*COL4A6*	−1.52	.0965	NS

DESeq2 differential-expression statistics (AF vs SR contrast) for the curated ECM genes highlighted in this study. Genes are grouped by functional category (basement membrane collagens, fibrinolysis inhibition, and interstitial collagen repair); for each gene, the log2FC, the Benjamini–Hochberg-adjusted *P* value (*P*_adj_), and a brief interpretation are provided. Positive and negative log2FC values denote up- and downregulation in AF, respectively. The complete 32-gene ECM panel, together with the per-gene detection counts, is provided in Table S1, Supplemental Digital Content 1.

AF = atrial fibrillation, ECM = extracellular matrix, log2FC = log2 fold change, NS = not significant, PAI-1 = plasminogen activator inhibitor-1, SR = sinus rhythm.

**Table 3:** Top 20 differentially expressed genes ranked by adjusted *P* value.

Rank	Gene	log2FC	*P*_adj_	Direction
1	*TRIM55*	+5.67	2.5e−12	Up
2	*GLI3*	−1.48	4.47e−12	Down
3	*HOXA4*	−2.95	4.47e−12	Down
4	*CDH19*	+4.45	2.27e−11	Up
5	*HOXA3*	−2.99	5.45e−10	Down
6	*GPR50*	−22.22	5.65e−10	Down
7	*LHX9*	+7.82	1.56e−09	Up
8	*MEIS1*	−1.78	1.56e−09	Down
9	*COL4A4*	+4.37	2.72e−09	Up
10	*GATA4*	+4.25	4.13e−09	Up
11	*ENSG00000255583.2*	+5.63	9.34e−09	Up
12	*ENSG00000233627.2*	−3.47	2.25e−08	Down
13	*MLIP*	+3.00	3.51e−08	Up
14	*TBX20*	+5.43	4.56e−08	Up
15	*TCF21*	+4.52	1.71e−07	Up
16	*CXXC4*	−2.21	4.88e−07	Down
17	*TULP3*	−0.97	1.55e−06	Down
18	*QSOX1*	+1.17	2.95e−06	Up
19	*PCDHAC1*	+2.83	3.63e−06	Up
20	*ENSG00000287538.1*	+5.15	3.63e−06	Up

The 20 most significant differentially expressed genes between AF and SR epicardial adipose tissue, ranked by Benjamini–Hochberg-adjusted *P* value (*P*_adj_). The columns are rank, gene symbol, log2FC, adjusted *P* value (*P*_adj_), and direction of change (relative to SR). Genes are given by the HUGO Gene Nomenclature Committee (HGNC) symbol; entries beginning with “ENSG” are Ensembl gene identifiers for transcripts without an approved gene symbol.

AF = atrial fibrillation, log2FC = log2 fold change, SR = sinus rhythm.

### 3.3. GSEA validation: ribosome biogenesis dysregulation

To validate the robustness of the core pathway, we employed GSEA using GO Biological Process gene sets (C5, homologous to MSigDB C5 GO:BP). Results consistently pointed to ribosome biogenesis as the most strongly dysregulated pathway family in AF-related EAT. One hundred thirty gene sets passed the significance threshold (FDR < 0.05), dominated by a coherent block of ribosome biogenesis, rRNA processing, mRNA splicing and processing, translation, and protein-folding pathways (NES = 2.0–3.0, FDR = 0.013); ribosome biogenesis and rRNA processing showed the strongest enrichment (NES = 3.03 and 2.83, respectively). In contrast, collagen fibril organization trended toward negative enrichment (NES = −1.57, FDR = 0.12), consistent with the suppression of interstitial collagen transcripts (Fig. 3, Table 4).

**Table 4:** Summary of gene set enrichment analysis results (GO biological process and KEGG gene sets).

Pathway/gene set	Size	NES	Nominal *P*	FDR
GO:0042254:ribosome biogenesis	121	3.03	.000144	0.013
GO:0006364:rRNA processing	161	2.83	.000139	0.013
GO:0006412:translation	295	2.47	.000127	0.013
GO:0042274:ribosomal small subunit biogenesis	75	2.76	.000151	0.013
GO:0042273:ribosomal large subunit biogenesis	39	2.62	.000161	0.013
KEGG:ribosome	173	2.26	1.37 × 10^−4^	0.012
KEGG:ribosome biogenesis in eukaryotes	78	2.64	1.49 × 10^−4^	0.012
GO:0008380:RNA splicing	308	2.32	.000127	0.013
GO:0006457:protein folding	178	2.28	.000136	0.013
GO:0030199:collagen fibril organization	64	-1.57	.00896	0.121

Leading enriched gene sets from GSEA of the AF-versus-SR comparison (fgsea: 10,000 gene-set permutations; DESeq2 Wald-statistic-ranked gene list). The columns are pathway/gene set (GO biological process or KEGG identifier and name), set size, NES, nominal *P* value, and Benjamini–Hochberg false discovery rate. A positive NES indicates enrichment in AF and a negative NES enrichment in SR; gene sets are ordered by NES.

FDR = false discovery rate, GO = Gene Ontology, GSEA = gene set enrichment analysis, KEGG = Kyoto Encyclopedia of Genes and Genomes, NES = normalized enrichment score, rRNA = ribosomal RNA.

**Figure 3.**
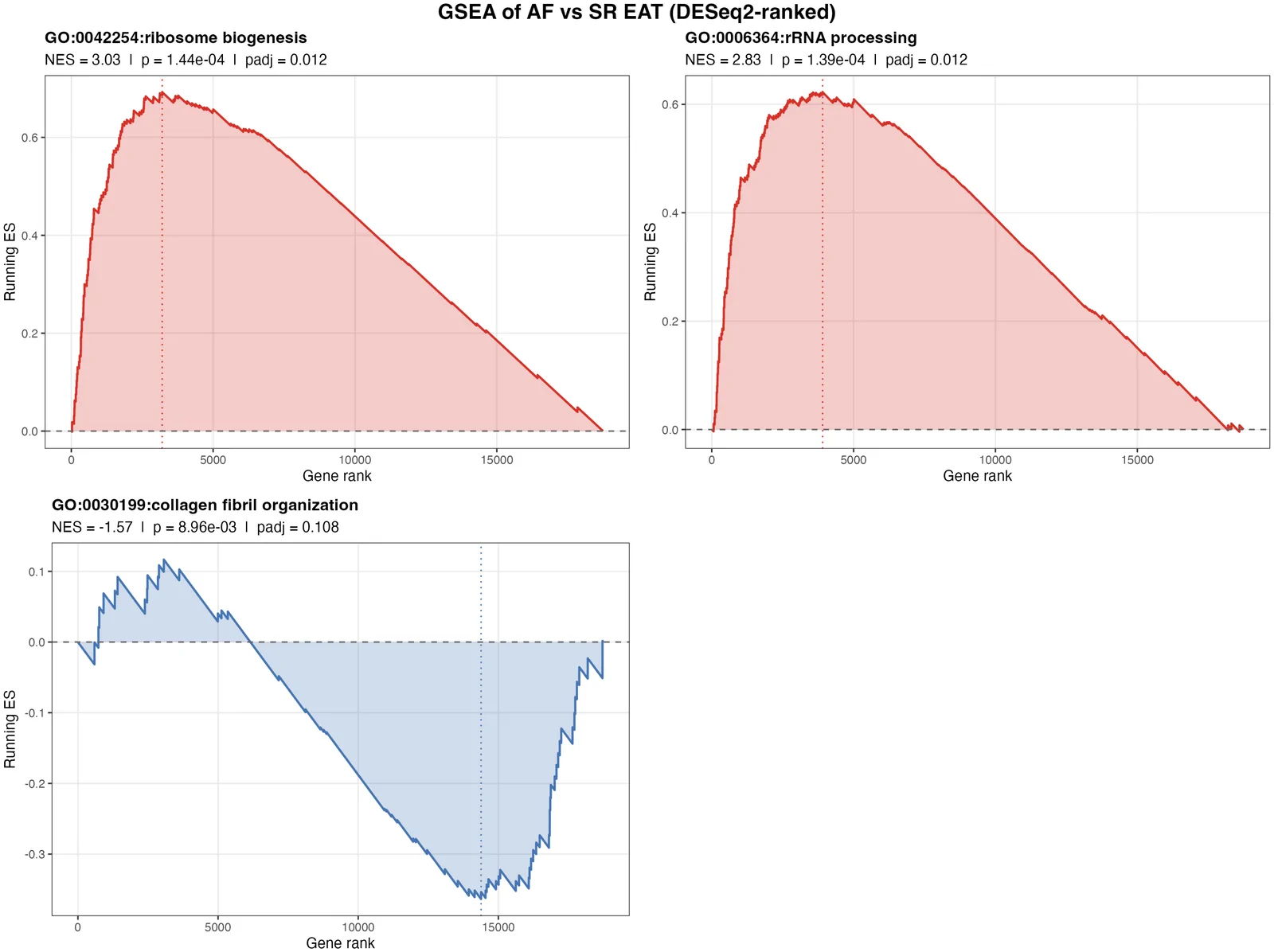
GSEA of AF versus SR EAT. GSEA was performed on the DESeq2 Wald-statistic-ranked gene list using GO Biological Process gene sets (fgsea, 10,000 gene-set permutations). (A) Ribosome biogenesis (NES = 3.03, nominal *P* = 1.4 × 10^−4^, FDR = 0.013). (B) rRNA processing (NES = 2.83, nominal *P* = 1.4 × 10^−4^, FDR = 0.013). (C) Collagen fibril organization, trending toward negative enrichment (NES = −1.57, nominal *P* = 9.0 × 10^−3^, FDR = 0.12). AF = atrial fibrillation, EAT = epicardial adipose tissue, FDR = false discovery rate, GO = Gene Ontology, GSEA = gene set enrichment analysis, NES = normalized enrichment score, rRNA = ribosomal RNA, SR = sinus rhythm.

The convergence of ribosome biogenesis enrichment with the ECM phenotype switch pattern suggests that ribosome biogenesis dysregulation may represent an associated upstream program (hypothesis-generating): impaired ribosomal function could compromise protein synthesis capacity in collagen-producing cells, potentially contributing to the preferential downregulation of interstitial repair genes, while basement membrane genes escape this suppression through independent regulatory mechanisms.

### 3.4. Mechanistic model construction

Based on these findings, we propose a mechanistic model of ECM phenotype switch in EAT during AF (Fig. 4). In the proposed model, ribosome biogenesis dysregulation acts as an upstream driver, impairing protein synthesis capacity in collagen-producing cells. This leads to a coordinated suppression of interstitial collagen repair genes (*COL1A1*, *COL1A2*, *COL3A1*, and *FN1*) while basement membrane collagen COL4A4 is independently upregulated through alternative regulatory mechanisms. Concomitant upregulation of SERPINE1 (PAI-1) further indicates enhanced inhibition of fibrinolysis within the remodeling matrix. The resulting imbalance – interstitial repair inactivation coupled with basement membrane deposition increase – represents the ECM phenotype switch that promotes atrial fibrosis and AF perpetuation.

**Figure 4.**
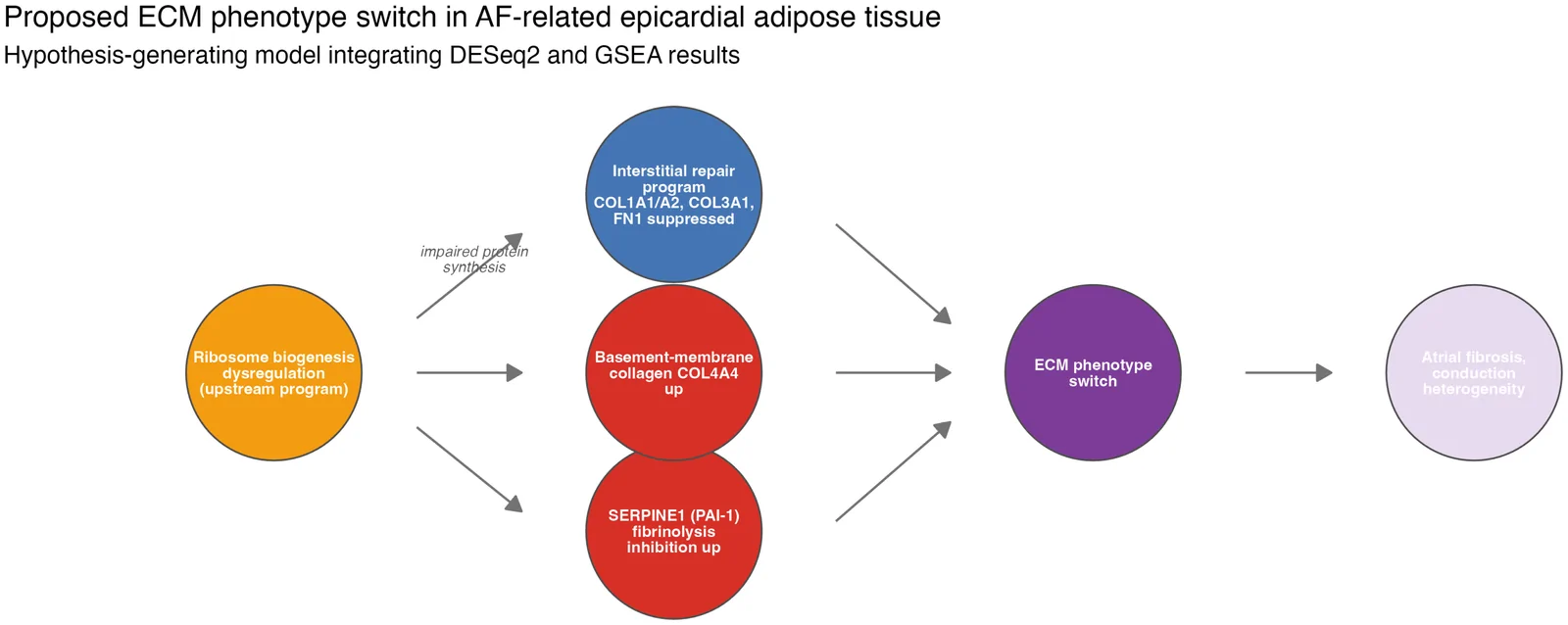
ECM phenotype switch mechanistic model in AF-related epicardial adipose tissue. Ribosome biogenesis dysregulation (hypothesized upstream program) is associated with suppression of interstitial collagen repair genes (*COL1A1*, *COL1A2*, *COL3A1*, and *FN1*) and independent upregulation of basement membrane collagen COL4A4, alongside upregulation of SERPINE1 (PAI-1, fibrinolysis inhibition). This imbalance drives basement membrane deposition, fibrosis, conduction abnormalities, and ultimately AF perpetuation. Key statistics: COL4A4 *P*_adj_ = 2.7 × 10^−9^; interstitial genes collectively downregulated (*P* < .05). AF = atrial fibrillation, EAT = epicardial adipose tissue, ECM = extracellular matrix.

## 4. Discussion

### 4.1. Biological significance of the ECM phenotype switch

Traditional views hold that AF-related atrial fibrosis is primarily characterized by excessive deposition of interstitial collagens (types I and III), leading to increased myocardial stiffness and heterogeneous electrical conduction.^[14]^ However, our study found that in AF-related EAT, not only are interstitial collagen repair programs suppressed, but basement membrane collagen COL4A4 is also independently upregulated, suggesting a more complex regulatory pattern of ECM remodeling.

COL4A4 is the alpha-4 chain of type IV collagen, primarily located in basement membranes, where it maintains basement membrane integrity and barrier function.^[15]^ The basement membrane is an important component of the ECM that separates epithelial/endothelial tissues from the interstitium and regulates cell polarity, proliferation, and differentiation. Upregulation of COL4A4 may lead to abnormal basement membrane structure, disruption of tissue barrier function, and promotion of inflammatory cell infiltration and fibrosis progression.^[16]^

The marked upregulation of SERPINE1 (PAI-1) adds a complementary dimension to this pattern: enhanced inhibition of fibrinolysis may, jointly with reduced collagen-synthesis transcripts, reshape the interstitial matrix, and PAI-1 upregulation is consistent with the profibrotic, proinflammatory milieu described in AF-related EAT.

Our proposed “ECM phenotype switch” hypothesis suggests that in AF-related EAT, these transcriptomic findings should be interpreted within the broader clinical evidence linking EAT inflammation and metabolic dysfunction to AF initiation, substrate formation, and recurrence after rhythm-control interventions. Inflammatory activity in periatrial tissues contributes to atrial fibrosis, electrical heterogeneity, and persistence of the arrhythmogenic substrate, and inflammation- and nutrition-related biomarkers have been associated with subsequent atrial arrhythmias and AF recurrence. For example, malnutrition assessed by the controlling nutritional status score was independently associated with long-term atrial high-rate episodes, AF, and all-cause mortality in octogenarians with dual-chamber pacemakers,^[17]^ and the uric acid-to-albumin ratio independently predicted AF recurrence after cryoballoon catheter ablation.^[18]^ Within this framework, the ECM-related transcriptomic alterations observed in the present study may reflect inflammatory and metabolic remodeling of EAT that contributes to AF maintenance and postablation recurrence, rather than representing a discrete causal phenotype switch.

### 4.2. Ribosome biogenesis as an upstream driver

Our study found that GSEA consistently identified ribosome biogenesis as the most significantly enriched pathway in AF-related EAT. Ribosome biogenesis is the rate-limiting step of cellular protein synthesis, and its dysregulation has been associated with various diseases, including cancer.^[19,20]^ Although current direct evidence linking ribosomal dysfunction to atrial fibrosis remains limited, the concept of ribosomal stress as a driver of cellular remodeling is conserved across multiple cell types and may drive the ECM phenotype switch through mechanisms including protein synthesis imbalance, activation of cellular stress responses, and metabolic reprogramming.

The convergence of our single-gene analysis (showing COL4A4 upregulation and interstitial collagen suppression) with GSEA results (ribosome biogenesis enrichment) supports a model where ribosomal dysfunction preferentially affects highly expressed structural proteins such as fibrillar collagens, while allowing basement membrane genes to be maintained or even upregulated through compensatory mechanisms. Future experimental studies are needed to validate this causal relationship.

### 4.3. Clinical translational implications

The ECM phenotype switch and ribosome biogenesis dysregulation identified in this study provide potential targets for AF prevention and treatment. COL4A4 may serve as a biomarker reflecting EAT fibrosis status and predicting AF risk. Ribosome biogenesis inhibitors such as CX-5461 have demonstrated antiproliferative activity in oncology studies^[21]^; their translational application in cardiovascular disease remains at a theoretical exploration stage and may exert antifibrotic effects by restoring ECM homeostasis, but requires further preclinical validation.

### 4.4. Study limitations

This study has several limitations. First, as a bioinformatics study based on a public database, it lacks an independent validation cohort. Second, the GSE135445 dataset has a small sample size (n = 12), limiting statistical power and generalizability. Third, because of the gene expression detection threshold, only 9 of 32 curated ECM genes were detected, and the analysis relied on single-gene statistics rather than module-level inference. Fourth, this is a cross-sectional analysis, and causality between the ECM phenotype switch and AF cannot be established. Sixth, gene-level counts were regenerated from the raw sequencing reads with an independent quantification pipeline (salmon/GENCODE v33); although all headline findings were robust to this requantification, exact expression values may differ slightly from the submitter-provided FPKM table. Seventh, functional experimental validation is lacking; the mechanism by which ribosome biogenesis dysregulation drives the ECM phenotype switch requires in vitro and in vivo experimental confirmation.

### 4.5. Summary and future directions

This study is the first to identify an ECM phenotype switch in AF-related EAT, characterized by suppressed interstitial collagen repair coupled with upregulated basement membrane collagen COL4A4, with ribosome biogenesis dysregulation as a potential upstream driver. This finding deepens our understanding of EAT’s role in AF-related atrial fibrosis and provides new potential targets for AF prevention and treatment. Future studies should validate the robustness of the ECM phenotype switch in independent cohorts and elucidate its causal mechanisms through cellular experiments and animal models.

## 5. Conclusion

Through single-gene analysis and GSEA, this study reveals an ECM phenotype switch in EAT during AF: interstitial collagen repair genes (*COL1A1*, *COL1A2*, *COL3A1*, and *FN1*) are downregulated, COL4A4 (basement membrane collagen) is independently upregulated, SERPINE1 (PAI-1) is upregulated, and ribosome biogenesis dysregulation is implicated as a potential associated upstream program. These findings provide a novel perspective for understanding EAT-mediated atrial remodeling in AF and offer potential intervention targets for AF prevention and treatment.

## Author contributions

**Data curation:** Huanmei Du, Shirui Li.

**Software:** Huanmei Du.

**Writing **–** original draft:** Huanmei Du.

**Formal analysis:** Huanmei Du.

**Supervision:** Xu Yang, Jianghua Zhang.

**Validation:** Xu Yang, Zukela Tuerhong, Jianghua Zhang.

**Visualization:** Yankai Guo.

**Conceptualization:** Jiasuoer Xiaokereti.

**Project administration:** Yujiao Zhang.

**Methodology:** Jianghua Zhang.

**Resources:** Jianghua Zhang.

**Writing – review & editing:** Xu Yang.




