# Supplementary material for: Mechanistic study of ECM phenotype switch in epicardial adipose tissue in atrial fibrillation: A bioinformatics study
Source: Medicine (Baltimore). 2026 Nov 6;105(45):e51461. doi: 10.1097/MD.0000000000051461 (PMC13747424; doi:10.1097/MD.0000000000051461)
Supplement: Supplementary file 1 [file medi-105-e51461-s001.docx]

Gene	Category	in_matrix	n_detected	detected
COL1A1	Interstitial repair	TRUE	12	TRUE
COL1A2	Interstitial repair	TRUE	12	TRUE
COL3A1	Interstitial repair	TRUE	12	TRUE
COL5A1	Interstitial repair	TRUE	12	TRUE
COL5A2	Interstitial repair	TRUE	12	TRUE
FN1	Interstitial repair	TRUE	12	TRUE
POSTN	Interstitial repair	TRUE	12	TRUE
VIM	Interstitial repair	TRUE	12	TRUE
ACTA2	Interstitial repair	TRUE	12	TRUE
TAGLN	Interstitial repair	TRUE	12	TRUE
MMP2	Interstitial repair	TRUE	12	TRUE
MMP9	Interstitial repair	TRUE	12	TRUE
MMP14	Interstitial repair	TRUE	12	TRUE
TIMP1	Interstitial repair	TRUE	12	TRUE
TIMP2	Interstitial repair	TRUE	12	TRUE
TGFB1	Interstitial repair	TRUE	12	TRUE
TGFB2	Interstitial repair	TRUE	12	TRUE
TGFB3	Interstitial repair	TRUE	12	TRUE
SERPINE1	Interstitial repair	TRUE	12	TRUE
SPARC	Interstitial repair	TRUE	12	TRUE
COL4A1	Basement membrane	TRUE	12	TRUE
COL4A2	Basement membrane	TRUE	12	TRUE
COL4A3	Basement membrane	TRUE	11	TRUE
COL4A4	Basement membrane	TRUE	12	TRUE
COL4A5	Basement membrane	TRUE	12	TRUE
COL4A6	Basement membrane	TRUE	12	TRUE
LAMA2	Basement membrane	TRUE	12	TRUE
LAMA4	Basement membrane	TRUE	12	TRUE
LAMB1	Basement membrane	TRUE	12	TRUE
LAMB2	Basement membrane	TRUE	12	TRUE
NID1	Basement membrane	TRUE	12	TRUE
HSPG2	Basement membrane	TRUE	12	TRUE
