# Supplementary material for: Mechanistic study of ECM phenotype switch in epicardial adipose tissue in atrial fibrillation: A bioinformatics study
Source: Medicine (Baltimore). 2026 Nov 6;105(45):e51461. doi: 10.1097/MD.0000000000051461 (PMC13747424; doi:10.1097/MD.0000000000051461)
Supplement: Supplementary file 2 [file medi-105-e51461-s002.docx]

dropped sample	Gene	log2FoldChange	pvalue	padj
GSM4009115	COL1A1	-1.815937946	0.0198141477651832	0.203675228046074
GSM4009116	COL1A1	-1.446542509	0.0586781134157313	0.348873967501866
GSM4009117	COL1A1	-1.11934345	0.11874377548128	0.490486152831922
GSM4009118	COL1A1	-1.550501093	0.0402490506187236	0.33130693940578
GSM4009119	COL1A1	-1.542645164	0.0378210178766003	0.249416665867238
GSM4009120	COL1A1	-1.520451943	0.0373437661632233	0.310417072251368
GSM4009121	COL1A1	-1.946332447	0.00808277848402957	0.13945058396714
GSM4009122	COL1A1	-1.948209841	0.00810535806124634	0.133494597106316
GSM4009123	COL1A1	-1.754784532	0.0245443418693084	0.212885532370177
GSM4009124	COL1A1	-1.93920939	0.00925763631552421	0.12655122983556
GSM4009125	COL1A1	-2.209202091	0.00287146174252849	0.0612306005737748
GSM4009126	COL1A1	-1.949832147	0.0380412685448725	0.171944017773462
GSM4009115	COL1A2	-0.925871505	0.0794469881540179	0.378550541333863
GSM4009116	COL1A2	-0.885606651	0.0927250145323143	0.428950165391207
GSM4009117	COL1A2	-0.485295355	0.27740728328466	0.676140211539375
GSM4009118	COL1A2	-0.822472449	0.10855566173787	0.504032532183166
GSM4009119	COL1A2	-0.788015909	0.107639378017415	0.415729304921757
GSM4009120	COL1A2	-0.812453582	0.107687046737482	0.486880405339105
GSM4009121	COL1A2	-1.109801314	0.0233444991063622	0.226625751852997
GSM4009122	COL1A2	-1.090850187	0.0298447125535323	0.247590529497132
GSM4009123	COL1A2	-0.988757044	0.0593992215434232	0.321250559510673
GSM4009124	COL1A2	-1.075837204	0.0346768006444439	0.241748534327296
GSM4009125	COL1A2	-1.130783085	0.0268343029185577	0.187785276599121
GSM4009126	COL1A2	-1.161889131	0.0205743682000057	0.11832021203512
GSM4009115	COL3A1	-0.755442604	0.221726243143073	0.588243598038201
GSM4009116	COL3A1	-0.707900143	0.251858589256905	0.640389929121028
GSM4009117	COL3A1	-0.158331098	0.761080925658364	0.934915033186954
GSM4009118	COL3A1	-0.629340727	0.295647422878521	0.716693355281062
GSM4009119	COL3A1	-0.738355909	0.226399021315084	0.57946673667384
GSM4009120	COL3A1	-0.556909406	0.31134168567368	0.71830392458577
GSM4009121	COL3A1	-0.9545401	0.0958169603986675	0.423861860431383
GSM4009122	COL3A1	-0.94229472	0.10657627252534	0.434726337354897
GSM4009123	COL3A1	-0.915731616	0.131154441974228	0.463298454115215
GSM4009124	COL3A1	-0.93387483	0.113345635761343	0.42614104360579
GSM4009125	COL3A1	-1.025369439	0.084862950646618	0.333387182609103
GSM4009126	COL3A1	-0.779111224	0.205411285696524	0.458037564642969
GSM4009115	COL4A4	4.38164807377004	3.94706469807779e-11	1.08193429001755e-0[illegible]
GSM4009116	COL4A4	4.28616692470825	8.36562524111428e-11	2.064636309507e-07
GSM4009117	COL4A4	4.24112813547114	7.72414036110062e-11	2.17383056895908e-0[illegible]
GSM4009118	COL4A4	4.33674768704644	6.23380680828786e-11	1.97868820353567e-0[illegible]
GSM4009119	COL4A4	4.6217981334339	1.71233773828884e-28	3.6758470476138e-24
GSM4009120	COL4A4	4.48846867262582	4.27606032486599e-12	1.31900425795898e-0[illegible]
GSM4009121	COL4A4	4.63019042359843	5.94325866540763e-13	2.81762269045872e-0[illegible]
GSM4009122	COL4A4	4.18448773507517	2.81653006732535e-11	6.93964843288293e-0[illegible]
GSM4009123	COL4A4	4.22438954317337	1.00649517167888e-10	1.79969078966735e-0[illegible]
GSM4009124	COL4A4	4.53140499994591	5.09812372844082e-12	1.78011914529358e-0[illegible]
GSM4009125	COL4A4	4.18741588346331	1.38801109904256e-10	2.77232083515435e-0[illegible]
GSM4009126	COL4A4	4.30492598326002	6.62083443541121e-11	6.26483726076872e-0[illegible]
GSM4009115	FN1	-1.310319588	0.0164383746129614	0.185189621588185
GSM4009116	FN1	-1.41049964	0.00451283107917687	0.104874454834355
GSM4009117	FN1	-0.776383622	0.109018517485624	0.475205917465294
GSM4009118	FN1	-1.075024248	0.0434375523604368	0.342549617108252
GSM4009119	FN1	-1.123960525	0.037338872513089	0.247779276914233
GSM4009120	FN1	-1.145683777	0.0358195774796269	0.305431829117054
GSM4009121	FN1	-0.945057992	0.0707792139458168	0.372122854659771
GSM4009122	FN1	-1.384142873	0.00763351275935964	0.128194724905131
GSM4009123	FN1	-1.425223847	0.00740630962923953	0.119548387539398
